# New Therapeutic Advances in the Management of Tricuspid Valve Regurgitation

**DOI:** 10.3390/jcm13164599

**Published:** 2024-08-06

**Authors:** Andreas Synetos, Nikolaos Ktenopoulos, Odysseas Katsaros, Konstantina Vlasopoulou, Theofanis Korovesis, Maria Drakopoulou, Anastasios Apostolos, Leonidas Koliastasis, Konstantinos Toutouzas, Constantinos Tsioufis

**Affiliations:** 1 First Department of Cardiology, National and Kapodistrian University of Athens, Hippokration General Hospital of Athens, 11527 Athens, Greece; nikosktenop@gmail.com (N.K.); odykatsaros@gmail.com (O.K.); vlasopouloukon@gmail.com (K.V.); faniskorovesis@gmail.com (T.K.); anastasisapostolos@gmail.com (A.A.); lkoliastasis@gmail.com (L.K.);; 2 School of Medicine, European University of Cyprus, Egkomi 2404, Cyprus

**Keywords:** tricuspid valve, tricuspid regurgitation, tricuspid transcatheter edge-to-edge repair, transcatheter tricuspid valve replacement, transcatheter heart valve procedures, transcatheter tricuspid valve procedures

## Abstract

Tricuspid regurgitation (TR) is an intricate disorder that has a negative outlook, while surgical treatment is linked to increased mortality. Primary TR occurs due to a structural defect in the tricuspid valve (TV), while secondary TR is a more prevalent condition often associated with pulmonary hypertension, heart failure, and atrial fibrillation. The use of specific surgical procedures to improve TR is limited in everyday clinical practice due to the heightened surgical risk and delayed patient presentation. The development of other transcatheter heart valve procedures has led to a significant increase in transcatheter TV operations, which can be attributed to certain technological advancements. This review aims to provide an updated overview of transcatheter TV procedures, available alternative therapies, and standards for patient selection. It will also highlight the current state of development in this field, which is characterized by rapid progress and numerous ongoing clinical trials.

## 1. Introduction

With the introduction of the initially developed transcatheter heart valve (THV) and subsequent improvements in technology, there has been a rising attentiveness in valvular heart disease (VHD), which has an expanding clinical effect and substantial financial impact. The main factor contributing to the surge of VHD is the growing lifespan of individuals. Tricuspid valve regurgitation (TR) is a condition that has historically been understudied and often undertreated compared to other valvular diseases like mitral or aortic valve disorders. For this reason, it is often called by many the “forgotten” valve. In recent years, however, innovation has focused on TR, mostly in order to allow for the reversibility of atrial fibrillation, bleeding risks, and multiorgan dysfunction, which are very common consequences seen in chronic right heart failure [1,2,3]. Patients seek definitive valve intervention when symptoms of dyspnea, fatigue, and edema do not respond to medical therapy, and surgical tricuspid valve (TV) repairs and replacements have traditionally been the main interventions, but their benefits compared to medical therapy are uncertain and they carry significant peri-procedural risks. Isolated TV surgery has a high in-hospital mortality rate of 9–10%, which is largely due to delayed intervention in highly symptomatic patients who have multiple comorbidities and right heart dysfunction [4,5,6].

In the contemporary era of interventional cardiology, transcatheter TV interventions (TTVI) have the potential to reduce the acute procedural and in-hospital adverse outcomes associated with traditional cardiac surgery. With a large, underserved patient population and early reports indicating safe and effective outcomes from TTVI, there has been a rapid development of devices to treat TR. Whether TTVI will improve survival rates compared to medical therapy is currently being investigated in ongoing randomized control trials.

The aim of this review is to outline the currently available techniques for the treatment of tricuspid regurgitation. Additionally, the available outcome data from TTVI may provide insights into what can be expected from the current pivotal trials.

## 2. Anatomical Considerations for TV

The TV consists of three leaflets of unequal size: the anterior, posterior, and septal leaflets. It is interesting to mention that this naming convention is based on the vertical orientation of the heart’s long axis, and when the TV is positioned in its anatomical orientation, where the long axis is rotated counterclockwise from vertical, the anterior leaflet is positioned anterior–superior, the posterior to inferior, and the septal leaflet is then posterior [7]. The anatomical position is crucial for guiding and determining the direction of catheters during transcatheter procedures [8].

Despite this, there is a traditional naming derived from the “surgical view” approach, which has long been used to facilitate clear communication between imaging specialists and surgeons, something essential for echocardiographically guided transcatheter interventions. In the most frequent type—the three-leaflet valve—the anteroseptal commissure is the longest, as the anterior and septal leaflets usually have the largest circumference [9]. There are also, typically, two distinct papillary muscles (anterior and posterior) and a third variable septal papillary muscle. The anterior papillary muscle is the largest, providing chordae to the anterior and posterior leaflets, originating from the anterior/lateral wall of the right ventricle (RV) near the trabeculations that include the moderator band [10].

From pathology studies, the number of TV leaflets that can vary in healthy individuals has long been recognized, but different terminologies have been used to describe these additional leaflets [11,12,13,14]. Another simplified nomenclature has been proposed recently that may assist in both pre-procedural planning and the execution of transcatheter devices, as well as assessments of procedural success [15,16,17]. Leaflet morphologies can be identified using either 2D transthoracic echocardiography (TTE) from modified views—as the TV is proximal to the anterior chest wall and diaphragm—or 2D transesophageal echocardiography (TOE) in the transgastric short-axis view, or their 3D volume-rendered equivalents [18].

The identification of additional leaflets is crucial and is performed by first considering deep indentations and true commissures as anatomically equivalent. Both leaflet folds and true commissures have more chordae along the leaflet edges, which can lead to potential sites for leaflet edge malcoaptation. Therefore, a separate leaflet is defined by an (i) independent motion from the adjacent leaflet and a (ii) color Doppler flow in systole, extending into the region around it.

There are four major classes of leaflet morphologies we are naming here: The classic three-leaflet morphology (Type I), the two-leaflet morphology with the fusion of the anterior and posterior leaflets (Type II), the four-leaflet configuration, with subcategories based on the location of the fourth leaflet (Type III), and morphology with more than four leaflets (Type IV) [18]. Leaflet structure and function can be used to further categorize TR into three types: Primary TR: Caused by pathological changes to the leaflets resulting in leaflet defects or malcoaptation. Atrial Secondary TR: Caused by insufficient leaflet coverage of a dilated tricuspid annulus (TA), associated with significant right atrial (RA) and TA dilation, less tethering or tenting of the tricuspid leaflets, and a normal or mildly dilated right ventricle (RV) with a more triangular shape and preserved function. Ventricular Secondary TR: Caused by insufficient leaflet coaptation due to apical displacement with leaflet tethering [19,20,21].

Although traditionally cardiac implantable electronic device (CIED) leads have been categorized as causing primary TR due to their direct effect on the leaflets or subvalvular apparatus, the presence of the device makes the resulting TR a complication secondary to the CIED leads [18].

Regarding the tricuspid annulus (TA), it is well known that the TV is a complex structure comprising interlinked components. These include leaflets hinged at the atrioventricular junction, chordae tendineae attached to the ventricular septum, or papillary muscles that arise from the ventricular wall. At the hinge of the leaflets, the leaflet surface might be overlapped with the RA myocardium (by 0.5–2 mm). The integrity of all the components of TV, optimal contraction of RA and RV walls, and the excursion of the orifice towards the apex, as well as an interaction with the left ventricle (LV), are the pillars of normal TV function. The anatomy of the fibrous TA is indistinct and incomplete, especially at the segment corresponding to the RV-free wall, which leads to potential dilation in these regions. Normally, the TA is almost oval and non-planar, but as the RV dilates, it becomes more circular. Thus, it is obvious that its geometry can be distorted if the RA and/or RV or the aortic root are dilated [22,23,24]. The TA is a dynamic compartment of the TV mechanism during the cardiac cycle, and interaction with leaflet coaptation affects the severity of TR and, therefore, patient prognosis [25,26]. The team of Addetia et al. measured with 3D TTE the size of the TA, demonstrating that it can change by about 30% between systole and diastole [27]. Ton-Nu and colleagues, using 3D-TTE as well, found that patients with significant secondary TR also had larger, more planar, and circular TAs compared to the controls [28], while Hirasawa and their team showed that an anteroposterior/septolateral ratio < 1.20 at the end-diastole was associated with greater RA and RV dilation, which further led to higher long-term mortality [29]. Additionally, the dimension of TA is affected by atrial fibrillation (AFib) with large beat-to-beat variations [30]. 

## 3. Classification of TR

TR used to be simply categorized based on leaflet involvement into primary (leaflet abnormalities) and secondary (normal leaflets with malcoaptation) disease. In the past years, they considered secondary TR as a distinct entity, affected by the structural integrity of the leaflets of TV and by RV remodeling as a result of pressure and/or volume overload, and CIED-induced TR has also been introduced as an entity [31]. Based on imaging features, Carpentier’s functional classification for a leaflet mobility-based description is used for the classification of secondary TR:

- Carpentier type I: Normal leaflet motion with predominant tricuspid annulus (TA) dilation (atrial secondary TR).
- Carpentier type IIIb: Leaflet tethering with restricted systolic motion (ventricular secondary TR).

Carpentier classification was originally designed to guide mitral valve surgery, and its usefulness for TR is less well-established [32]. The varied outcomes based on the etiology of secondary TR, along with multiple different morphological characteristics that predict a recurrence of TR post-surgical repair, have led to a redefinition of the classification of secondary TR based on primary cause, distinct pathophysiology, and characteristic imaging features, recognizing that “not all secondary TRs are the same”, including differences in TV leaflet mobility, coaptation, and characteristic differences in the remodeling and function of TA, RV, and RA [33,34,35,36]. Atrial secondary TR includes some morphologic elements, like leaflet tethering, RA dilatation with normal RV, proper LV function, and truancy of pulmonary hypertension, notwithstanding the presentation rhythm [37].

This contemporary classification of TR has important prognostic and treatment implications, discriminating between atrial and ventricular forms of secondary TR [33,38,39]. Atrial secondary TR progresses rapidly and has poor outcomes, with secondary RV dilation and/or RV dysfunction developing in later stages. Although evidence is limited, rhythm control in AFib may lead to a reduction in atrial secondary TR in some patients by the reverse remodeling of RA and TA [31,40]. 

Another important consideration is the incidence of CIED-induced TR that is caused by devices, which is expected to rise since there is an aging population, with more device implantations and inevitable complications [31,41,42]. CIED-related TR has a pathophysiology that shares characteristics of both primary and secondary TR. It also has unique epidemiology as well as distinct options for its management; CIED-related TR can be considered a third distinct category [8,43,44,45]. The wide range in the incidence of post-implantation TR is probably a matter of the difficulty in identifying the association between the wires/catheters and TV dysfunction using 2D echocardiography; however, the clinical implementation of 3D echocardiography allows better documentation of these pathophysiological relationships [46,47,48].

Therefore, patients with TR and a CIED can be categorized into primary and secondary categories. Primary CIED-induced TR can be defined as a two-grade increase in TR severity after CIED implantation [44]. Contrariwise, secondary CIED-induced TR is a result of the remodeling of the TV after the dilatation of RV caused by pacing or heart failure. Seo and colleagues reported that there was secondary origin in even 60% of worse-progressed TR post-CIED [49]. Predictors of secondary CIED-induced TR include permanent AFib, a history of open-heart surgery, as well as documented RV dilation in previous studies [49,50].

The clinical importance of diagnosing CIED-induced TR is underscored by its impact on long-term RV function and its association with poor outcomes [49,51,52,53,54,55]. Accordingly, in patients with compelling evidence of CIED-related severe TR based on clinical, hemodynamic, and echocardiographic assessments, corrective interventions should be promptly considered to prevent severe dilation of the TA and RV as well as severe dysfunction of the RV [56]. In patients with severe TR related to CIEDs undergoing consideration for lead extraction, identifying the type of lead interference through a 3D echo is crucial. This is because the procedure may exacerbate TR in cases involving perforated leaflets, chordae avulsion, or severe adherence or entanglement of leads.

## 4. Patient Selection

The most significant parameter to ensure the effectiveness of the TEER procedure is the proper selection of the population. The selection among various surgical and interventional options for treating TR should be guided by the underlying mechanism of regurgitation, patient conditions, and the etiology of the disease [35,57,58]. Evaluating the anatomical–functional aspects of the TV is crucial in deciding whether replacement or repair is chosen.

Multimodality imaging plays a pivotal role in selecting the optimal treatment strategy [59,60]. Especially in the era of interventional technologies allowing catheter therapies, careful anatomy-based device selection is crucial for achieving favorable outcomes. Mechanisms of regurgitation assessment should encompass annular, leaflet, and subannular components, which can be classified akin to Carpentier’s classification of mitral regurgitation, in order to facilitate communication [61,62].

In cases of functional TR, annular dilatation and leaflet tethering are the primary components of valve regurgitation. Depending on the predominant mechanism, corrective actions are undertaken to restore valve competence [61]. Valve replacement is favored for cases with progressed dysfunction where repair is deemed ineffective or unsustainable.

Surgery remains the gold standard treatment for symptomatic patients with severe TR [31]. Nevertheless, isolated tricuspid valve surgery (ITVS) is rarely performed due to its high mortality rates [63]. Those high mortality rates seem to be mainly attributable to the high perioperative risks of patients who are referred for surgical intervention too late. In the modern era of transcatheter intervention development, aiming for patients’ best care practice and using supportive clinical tools to guide the routine practice and decision-making for this heterogeneous group is of high importance. The most commonly used Society of Thoracic Surgeons (STS) cardiac surgery risk model and the logistic EuroSCORE/EuroSCORE II were not designed to apply to these types of interventions. Recently, three risk score models have been suggested to predict the outcome of patients after ITVS for severe TR. Those are the TRI-SCORE [63], the TRIO-SCORE [64], and the Novel Risk Score by Wang Tom Kai Ming et al. [65] (Table 1). The TRI-SCORE predicts in-hospital mortality between the intervention and hospital discharge during the same hospital stay, whereas the TRIO-SCORE predicts the 10-year all-cause mortality, and the Novel Risk Score predicts the 1-year all-cause mortality, with the first one having the highest predictive ability with a C-statistic value of >0.75 [63,64,65]. Moreover, although designed to predict in-hospital mortality, the TRI-SCORE also showed good prediction accuracy for in-hospital major complication rates and 1-year mortality [63]. Patients with lower risk scores and fewer comorbidities have been associated with worse survival rates, while TR severity is increasing. This means that early intervention could be beneficial for this proportion of patients, leading to lower mortality rates [64].

Surgery remains the gold standard treatment for low-risk patients with functional TR. A recent study developed a specialized risk score model to predict patient outcomes after isolated TV surgery (ITVS) for severe TR, incorporating parameters of age, New York Heart Association (NYHA) Class, signs of right-sided heart failure, diuretic use, kidney function, levels of bilirubin, LV ejection fraction, and RV dysfunction severity [63]. Annuloplasty is commonly performed; however, cases involving leaflet tethering can be challenging, with even less success when there is RV dysfunction and/or remodeling [66]. The management and repair of complex congenital anomalies (such as Ebstein) or TR caused by endocarditis necessitates a surgical approach [67,68]. In patients with functional TR and advanced disease undergoing surgery, valve replacement is preferred instead of repair, especially in modern times, given the high-risk profile of reinterventions, although evolving interventional algorithms may impact future choices [69].

Imaging remains pivotal in guiding decisions regarding interventional procedures, especially in cases of repair [70]. In most instances, interventions target the specific lesion with a single device addressing one specific element that has a dysfunction; contrasted with surgery, it is more common to see combined procedures. Over the last decade, numerous devices have been developed to replicate surgical techniques via catheterization. While many procedures are still investigational, some interventional approaches are increasingly prevalent, with transcatheter edge-to-edge repair (TEER) being the most extensively experienced [71]. The TEER approach is suitable for most patients; however, early data indicate that certain patient populations may not achieve an optimal reduction in the TR to ≤2+ or improved outcomes [15,17,72,73,74,75,76,77]. The success of tricuspid TEER has also been linked to valve morphology, with more complex morphologies associated with lower procedural success, potentially influencing overall outcomes. Moreover, RV ejection fraction is strongly correlated to outcomes following tricuspid TEER [76,77].

To avoid underestimating the severity of TR, it is recommended to use quantitative parameters derived from the Proximal Isovelocity Surface Area (PISA) method, such as Effective Regurgitant Orifice Area (EROA) and regurgitant volume. These metrics offer a more accurate assessment of TR severity than qualitative evaluations. Additionally, discussing the application of correction formulas to the PISA method can further enhance the precision of TR severity measurements [78].

Using a 3D vena contract area as an alternative can be advantageous, as this technique can improve the echocardiographic evaluation of TR severity and offer additional insights. A recent five-degree grading scheme for post-procedural TR assessment [79] provided a more detailed and accurate measure of post-intervention outcomes, potentially significantly enhancing the evaluation of procedural success.

Furthermore, elaborating on the importance of evaluating TV anatomy and the echocardiographic characteristics of the TR jet is crucial for achieving optimal results with transcatheter tricuspid edge-to-edge repair (T-TEER). The GLIDE scoring system can help predict procedural outcomes for T-TEER by assessing key anatomical and procedural factors, thereby guiding patient selection and improving procedural success [80].

Several annuloplasty devices are on the market and could be employed as standalone procedures, particularly in patients with less pronounced leaflet tethering. For patients with more advanced geometric alterations of the components of TV and the RV, valve replacement remains an option in its nascent stages, lacking commercially available devices. The patient selection primarily relies on CT scans to assess the dimensions of the TA, anatomy and size of the RV, and positioning of the vena cava. Echocardiography and right heart catheterization play crucial roles in patient selection to exclude individuals with excess right heart disease and elevated pulmonary resistance. Notably, 3D echocardiography demonstrates high repeatability when compared to CT in evaluating the morphology and dimensions of the TV annulus [31,81].

## 5. Transcatheter Tricuspid Valve Therapies

Nowadays, there are percutaneous procedures that imitate surgical approaches. Such alternatives are approved by the EU and include leaflet approximation devices, direct annuloplasty, and heterotopic caval valve implantation. Additionally, there are transcatheter TV replacement (TTVR) platforms for THV placement, but they are not yet accessible for ambiguous use. There is increasing research regarding the use of TTVI for individuals who are unable to undergo surgery or are at high risk for surgical procedures. In two groups of cases with similar characteristics, mortality rates were lower when TTVI was implemented instead of typical medical care, and there was also an amelioration in the likelihood of re-hospitalization for heart failure. Validation of these results through randomized controlled studies is necessary.

The 2021 European guidelines for the management of TR provide an IIb level C recommendation for the TTVI of severe TR in patients who are unable to undergo surgery. The recommendations also emphasize the significance of referring patients with TV disorders early and the necessity of addressing the TV disease in case of surgery in the LV.

### 5.1. Leaflet Approximation Devices

The Tricuspid Transcatheter Edge-to-Edge Repair (TEER) device is widely used globally. Initially, the MitraClip device was applied unauthorized, prior to the creation and certification of the PASCAL and TriClip platforms. A thorough TEE study before the operation for the assessment of the valve anatomy can help dictate if the patient is fit for the operation and there is a high chance of success when employing TEER techniques.

The shape, quantity, and position [17] of the TV components have an impact on the anatomic approach, clip location, the number of clips used, and the chance of a favorable procedural result [16]. Optimal depletion of TR is associated with specific features, including a small aperture between the TV leaflets, a major TR jet located centrally, and a TV with three leaflets [73]. When using previous clip appliances, coaptation gaps of 7.0 mm or more were shown to be related to significant excess TR at the finalization of the treatment [82]. However, using novel and more updated equipment with lengthier arms, a breach as broad as 10 mm can still be effectively handled [72,83].

Other variables that may influence individuals’ selections for TEER include pre-existing RV disarray, TR associated with CIED, and discrepancy for OACs [84]. The TEER device can be advantageous for persons with severe RV failure, as it can reduce TR without generating abrupt changes in the RV afterload. This is closely associated with the degree of TR after TTVI [44]. However, this advantage remains strictly theoretical as there is no documentation to support the idea that different component selections have any effect on the functioning of RVs. The presence of CIED leads might interfere with the correct positioning of the leaflets and reduce the efficiency of TEER in cases when the lead is directly causing TR to the TV [44]. In such situations, it is reasonable to consider removing or altering the lead and opting for an alternative CIED, such as implanting a lead in the coronary sinus or choosing a leadless option. Unfortunately, the effectiveness of lead extraction is still questionable and is probably influenced by the duration of time the lead has been inserted, as well as the precise approach used to block the lead. When determining the method, it is crucial to take into account the following factors: an assessment of the patient’s susceptibility to adverse effects and objectives for medical intervention [85].

#### 5.1.1. Triclip

**Figure 1:**
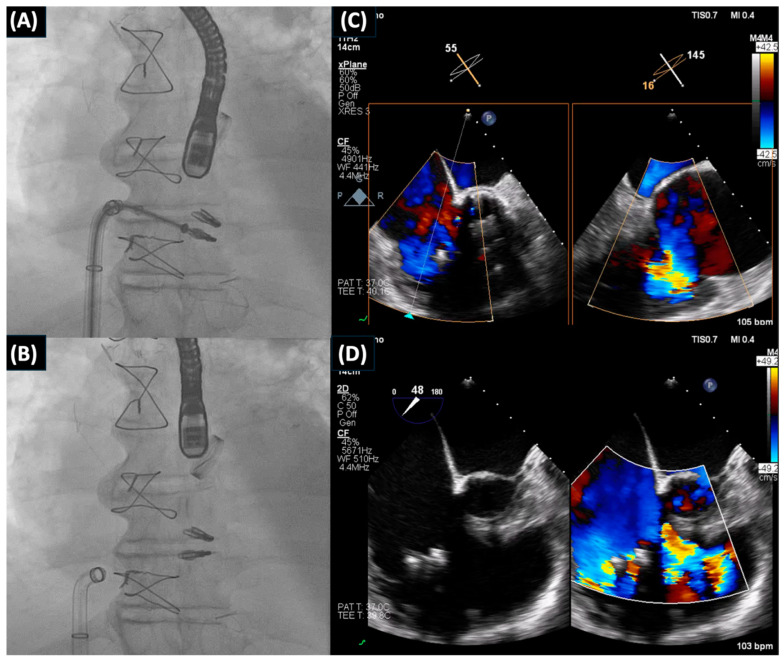
A 81 year old man presented with symptoms of RV heart failure and torrential tricuspid regurgitation. He was under optimal medical therapy. Echocardiography subsequently demonstrated torrential tricuspid regurgitation with posterior leaflet flail. After Heart Team review, the patient underwent transcatheter tricuspid valve repair with the TriClip™ system (Abbott Vascular, Santa Clara, CA, USA). (**A**,**B**): Transcatheter tricuspid valve repair with the TriClip™ system. (**C**,**D**): Intra-procedural transoesophageal echo revealing the torrential tricuspid regurgitation and assisting the operators for the optimal implantation of the clips.

The TriClip system diverges from the MitraClip in that it has the capacity to move opposite to the septum, along with a stubby guide catheter. TEER with the TriClip platform is commonly carried out with general anesthesia. It is led by TEE and involves the utilization of a 24 Fr catheter through the common femoral vein and IVC. The TriClip comes in different sizes, which vary in extent and breadth, and include NT (normal length and width), NT-W (extra broad), XT (extra extensive), and XT-W (extremely extensive and broad) [86]. A personalized technique is employed to ascertain the suitable dimensions of the system. According to the TRILUMINATE study, which assessed 85 individuals with a primary endpoint at one year, 86% of them had a decrease in TR of at least one proportion [87]. Moreover, 70% of cases retained a moderate or lower degree of TR, and notably, 56% of individuals with severe or excessive TR also achieved this level of improvement [88]. Notable enhancements in echocardiographic and objective measures were noted, and these benefits were sustained at the one-year mark after achieving the 30-day one. The rate of single leaflet device adjunction was 8%, with no instances of device embolization [87]. (Figure 1) Following the TRILUMICATE trial, the TRILUMINATE Pivotal trial randomized 350 individuals with severe TR to either TEER or medical therapy. The results favored TEER regarding its safety profile, while there was a reduction in TR severity and an improvement in quality of life through the 1-year follow-up. Extended data will provide information on the long-term sustainability of these findings, and additional studies may accurately determine which patients with TR will derive the greatest benefit from this intervention [89].

#### 5.1.2. Pascal

The PASCAL device comprises a 22 Fr TF catheter equipped with a system that includes an inner separator attached to two wide spades with grasps that may motion separately. The system is constructed from nitinol and has a passive completion mechanism that has the potential to minimize leaflet damage and tension. The updated PASCAL Ace device is designed with a slimmer and stubby shape, along with lengthier grasps, to effectively address the challenges associated with TEER procedures [86]. The initial effectiveness of the CLASP TR trial showed a prudent outline, with a decrease in TR of at least one degree in 85% of the 29 cases who underwent a fortunate procedure [90]. A total of 32% of the participants received two clips, and the average duration between the devices was 168 ± 152 min. After 1 month, there was a notable refinement in TR, with 52% experiencing moderate or fewer symptoms. Additionally, there were significant increases in functional capacity, exercise tolerance, and quality-of-life expedients [90].

#### 5.1.3. Mistral

This nitinol system, which has a spiral structure, enhances the contact between the components of the TV by bringing certain chordae aside without causing injury. This results in a configuration comparable to a “flower bouquet”, which lowers the distance between the chordae and the leaflets, promoting their alignment [86]. This TF percutaneous implantation utilizes an 8.5 Fr catheter to guide the delivery equipment into the RV. The system is available in two options: one with an exterior dimension of 8.8 mm and the other with an exterior dimension of 7.4 mm [86]. There have been seven recorded patients with operation times ranging from 58 to 125 min. All treatments were effective, as evidenced by the 30-day outcomes, which showed a considerable depletion in TR, an improvement in symptoms, and the absence of any adverse effects [91].

### 5.2. Annuloplasty Devices

Annuloplasty, a frequently used procedure for patients in need of heart surgery, has not yet been widely accepted as a percutaneous treatment option [86]. It primarily focuses on facing the functional TR caused by the enlargement of the TV annulus rather than the conjecture of the TV leaflets. In order to provide results similar to those achieved after surgery, the majority of percutaneous systems stretch from the antero-septal to the postero-septal commissures to create an irregular cylindrical form. In addition, it has the advantage of maintaining the original anatomical structure, which enables potential ensuing procedures, including leaflet modification or TVR, while it does not necessitate the use of OACs. Percutaneous annuloplasty devices may be categorized into three groups: ring-based (either direct or indirect), suture-based, and non-suture-based devices [86].

In general, early efficacy trials demonstrate sufficient operational safety and effectiveness. However, there are a few significant technical difficulties and unique issues that should be noted. Concomitant operational imaging is once again vital and can pose challenges in these operations [92]. Several direct annuloplasty systems need a rigorous and time-consuming procedure of consecutive annular anchoring. This, along with challenging imaging, leads to excessively long operational timeframes, typically averaging close to 5 h [92]. The main annuloplasty devices are presented below:

- The Cardioband Tricuspid Repair System is a band that is designed to mimic the surgical MV system. It is sutureless, customizable, and applied in a straight manner [93,94].
- The DaVingi TR system is a two-step deployed device using a 22 Fr sheath to insert an annuloplasty ring into the jugular vein and put it on the annulus from the atrial side. Anchors secure this to the annulus by being concurrently deployed [95].
- Transatrial intrapericardial tricuspid annuloplasty is a method of repairing the TV by inserting a reusable annuloplasty apparatus into the pericardial cavity through a puncture in the right atrial branch [96].
- The Minimally Invasive Annuloplasty (MIA) approach employs compact, polymeric hooks and a thermoplastic polymer to offer compression to the hooks [86].
- PASTA, which stands for pledget-assisted suture tricuspid annuloplasty, is a procedure that includes using pledgets to reinforce sutures in the TV. In this method, two pledged strands are passed through both the septal and lateral annulus, with each strand having two incisions. This approach is used to optimize the strength and is a distinguishing feature of PASTA [97].
- Transcatheter Alfieri stitch for tricuspid insufficiency (TASTI) refers to a medical procedure that is performed using a technique known as transapical leaflet traversal. This method involves the use of guidewires to pass through both the septal and lateral leaflets. Subsequently, the guidewires are captured and substituted with stitches that incorporate pledgets on the side of the RA [86].
- The K-Clip transjugular system employs an external divert sheath that is placed in the RA. An inner divert sheath carrying a clip is inserted via this outer sheath. The clip is placed into the annulus, running perpendicular to it, and positioned between the leaflets, while a catheter protects the RCA [98].

### 5.3. Coaptation Enhancement Devices (Spacers)

These systems are specifically built with a central spacer that improves the alignment of the leaflets and decreases the size of the region where regurgitation occurs. The spacer is connected to a secondary element, which might be either an attached caval stent or an anchor that is firmly fixed inside the heart. This equipment has several advantages, including the ability to handle a wide variety of annulus widths and address significant coaptation gaps. In addition, they refrain from engaging in direct contact with circular structures, thereby eliminating the need for intricate imaging and resulting in shorter device operation durations differentiated from alternative methods. Nevertheless, this approach does not permit re-operation, and further clinical results are still necessary. The two available devices are the CroiValve DUO and the Tripair.

### 5.4. Transcatheter Tricuspid Valve Replacement (TTVR) Systems

TTVR is emerging as a minimally invasive procedure for TV displacement that seeks to eliminate TR in contrast to leaflet approximation and annuloplasty. Procedural factors pertain to the formula of anchoring, the form and size of the annulus, the delivery technique, and the path of the IVC to the annulus. TTVR may potentially cause injury in people with limited RV capacity. Various TTVR systems possess distinct anchoring processes, resulting in varying imaging prerequisites. Typically, the imaging requirements for TTVR are lower compared to leaflet coaptation or annular systems [26,86,99].

TTVR devices can be percutaneously installed in individuals who have substantial gaps between the TV leaflets, limited movement of the leaflets, or TR associated with CIED. Remarkably, nonetheless, the CIED lead is positioned between the equipment and the heart wall, which complicates its extraction. OACs are now advised because of the higher probability of THV thrombosis in a low-pressure system in the RV, with the possibility of extra antiplatelet medication being taken into account. The current proportions of the available THVs are designed to accommodate only small circular dimensions, which is a significant limitation of TTVR now. The available TTVR systems are mentioned below:

1. The EVOQUE device is a TF THV that is made out of bovine pericardium and consists of three leaflets. The appliance is reinforced by a nitinol skeleton consisting of nine anchors and is enclosed by a cloth skirt [99]. The TRISCEND study, through the 1-year follow-up, revealed that there was a 9.1% rate of death for all causes and a 10.2% rate of hospitalization for heart failure. Additionally, 97.6% of patients witnessed a substantial reduction in TR, while there was a noticeable increase in functional capabilities and quality of life outcomes [100].
2. The LuX valve is a versatile delivery method comprising a bovine pericardial valve connected to a nitinol THV prosthesis. The device comprises an atrial disc, an interventricular septal anchor, and two clamps covered with expanded polymer [101].
3. TriSol is a bovine pericardium-based prosthetic THV. The structure comprises a solitary leaflet and is connected to two commissures, enabling it to operate as a bicuspid device. The THV is connected to a thin, self-expanding cylindrical framework made of nitinol. The design has a narrow center and six arms that hold the THV in position by grasping it within the current leaflets and the surrounding walls [101].
4. Intrepid is a circular apparatus that houses a TV constructed from bovine pericardium. The device is introduced into the human system either by a minor surgical cut in the thorax or via a vessel in the leg, using a catheter with a diameter of 35 French units [86].
5. Additional TTVR systems are now undergoing preclinical trials, and First-in-Man investigations include TriCares (Aschheim, Germany, NCT05126030), VDyne (Maple Grove, Minnesota), and CardioValve (Yehuda, Israel, NCT03958773) [86]. The Cardiovalve system was tested in an early feasibility study with 20 individuals with high rates of technical success, an acceptable rate of adverse events, and improvement in TR [102,103].
6. The TricValve is a TF device that consists of two self-expanding valves that are positioned in the inferior and superior vena cava [104].
7. The Tricento is a TF device that has a self-expanding nitinol stent frame. The design of this stent structure is specially tailored to be elongated and span from the superior SVC to a position above the hepatic vein. The pericardial tissue THV, which consists of two grooves, is positioned in such a way that it faces the RA when it is deployed [105].
8. Inferior caval valve implantation (CAVI) is a feasible and cost-effective procedure that involves placing a balloon-expandable valve in the transfemoral area, along with a self-expanding prosthesis in the IVC [106].

Table 2 summarizes the most important transcatheter tricuspid valve repair and replacement devices currently available.

## 6. Current Limitations and Future Perspectives

The emergence of TTVI is a direct response to the substantial unmet need resulting from the longstanding absence of adequate therapy for TR, even though the initial studies suggest their relative safety. In this setting, our understanding of the disease’s development and our ability to evaluate it, utilizing both non-invasive and invasive methods, has advanced [26,86].

TTVIs, either for repair or replacement, may occasionally fail to effectively treat TV conditions. Several constraints have been delineated. Occasionally, the TV may not be completely repaired following the operation, resulting in remaining TR. This treatment may have a possibility for advancement despite initial positive outcomes. TTVIs have the potential for procedural consequences, including the risk of major or minor bleeding and infection. In the event that these adverse events arise, they will have a significant influence on the relative effectiveness of the procedure. Although the topic of lifetime management is favored, the most successful approach would be to implement a single, long-lasting procedure. Ultimately, the effectiveness of TTVIs relies on the expertise of the interventional cardiologist and his/her team, as well as the patient’s specific health condition and any additional medical ambiances they may have.

Modifications to the existing clinical practice and beliefs for treating TR are essential, as they are mostly derived from experience and conceptions related to surgical procedures, left-sided THV interventions, and medical treatment, with an additional lack of standardized definitions. Delaying any potential intervention until each individual experiences serious repercussions of severe TR is not advisable. Considering the high level of safety associated with TTVI, particularly for TEER, it is necessary to start considering referral and transcatheter treatment at an earlier stage than what is currently happening.

These evaluation methods have the capability to improve the uniformity of TV disease diagnoses [8], severity criteria [79], and clinical results [107]. The categorization of TV terminology and recognition of TV structural changes [16] has been essential in this domain, particularly given the worldwide rise in instances of leaflet proximity. The recent use of the five-grade TR severity scale, encompassing the classifications of mild, moderate, severe, massive, and torrential, has gained significant recognition in prominent TV therapy research. The initiative is now included in the European guidelines [31]. The utilization of the enlarged grading technique in the evaluation of individuals for TTVI may yield a more precise assessment of operational appropriateness and the impact of a single drop in TR degree on clinical signs, endurance, and recurrent hospitalization due to heart failure. Furthermore, it enables a more accurate evaluation of the degree of benefit across various device substrates and is anticipated to ensure the utilization of a reliable, evidence-based approach for patients experiencing TR [86]. 

The area of TTVI is still in its nascent stage, and as recipients become more familiar with it, researchers are continuously investigating issues related to device selection and durability. Continued progress in the field of publicly available data and analysis will improve our comprehension of the most effective technology in terms of both safety and clinical results. Although seniors who are now receiving treatment have a high occurrence of serious disease and a short remaining lifespan, TTVI should aim to obtain comparable long-term results and avoid the need for further interventions, just like surgical operations. The simultaneous TTVI of TR during non-tricuspid treatments attempts to imitate the surgical strategy to address multiple valve diseases. Nevertheless, the advantage of TTVI resides in its ability to arrange these procedures in a certain order and perhaps reduce the necessity for unneeded operations [79,86].

Device makers continue to enhance their devices by addressing both real and perceived limitations of current technologies. Procedurally, there are notable drawbacks that parallel the difficulties seen during the initial phases of aortic and mitral procedures. At first, procedural durations are long; however, as operators become more familiar and proficient with these procedures and infrastructure, the durations appear to improve. Currently, the use of TEE for guidance during operations requires both the proceduralist and the imaging operator to possess expertise and knowledge. ICE plays a prominent role in structural intervention and has demonstrated progress in imaging. The utilization of innovative probes with the ability to do four-dimensional imaging has the potential to reduce the intensity of imaging and mitigate the problems associated with TTVI. In addition to improving imaging equipment, device manufacturers must also prioritize strengthening device safety. This includes creating smaller delivery systems and improving operator usability, as demonstrated by the advancements made with transcatheter aortic devices. Technological progress in delivery systems that allow for versatile movement in several directions will assist in resolving anatomical difficulties, such as the mismatch between the IVC and the TV annulus [86,88].

Considering the long-term treatment of patients with VHD is crucial since the necessity for intervention becomes more evident at an earlier stage. The incorporation of procedural “repeatability” and “durability” will have a notable influence on TV interventions, akin to their effect on TAVR. In theory, the use of TTVR and annuloplasty therapies will facilitate valve-in-device surgical procedures.

Ongoing advancements are being made in the management of TR in cases with structural heart disease. Nevertheless, obtaining an early diagnosis and referral is important to optimize outcomes [86].

It is important for primary care physicians and primary cardiologists to be knowledgeable about how to recognize symptomatic TR patients and comprehend the potential adverse outcomes that might result, even from mild disease.

By directing these patients to Level 1 Valve Centers [31], they will undergo essential diagnostic procedures to assess the extent of their problem, receive optimal medicinal treatment, and have the opportunity to participate in surgical procedures or trials for TTVI.

## 7. Conclusions

TTVI is experiencing significant expansion, allowing a broader population to qualify for TTVI for severe TR. The majority of the current TTVI devices appear to possess safe and efficacious characteristics and are typically capable of managing various TR causes. The current randomized clinical trials seek to address the issues of patient selection, device efficacy, and long-term outcomes. Given the growth of clinical practice and the progress of modern medical technologies, it is highly likely that TTVI will emerge as a free and beneficial treatment option for a group of patients who have been overlooked, like transcatheter aortic and mitral interventions.

## Figures and Tables

**Table 1:** Tricuspid valve surgery risk scores.

Risk Scores	TRI-SCORE [63]		TRIO-SCORE [64]		Novel Risk Score by Wang Tom Kai Ming et al. [65]	
	**Parameters**	***Risk Points***	**Parameters**	***Risk Points***	**Parameters**	***Risk Points***
	Age ≥ 70 years	1	Age 70–79 years ≥80 years	1 2	Age 65–74 years ≥75 years	1 2
	NYHA III–IV	1	Male sex	1	Myocardial infarction	1
	Right-sided HF signs	2	HR ≥ 90 beats/min	1	Peripheral vascular disease	1
	Furosemide ≥ 125 mg/day	2	Congestive heart failure	2	Loop diuretic use	1
	LVEF < 60%	1	Severe TR	1	RV systolic function: Mildly impaired Moderately impaired Severely impaired	1 2 3
	Moderate/severe RV dysfunction	1	Creatinine ≥ 2 mg/dL	2	RVSP > 50 mmHg	1
	GFR < 30 mL/min	2	AST ≥ 40 U/L	1	CKD (creatinine > 1.4 mg/dL)	1
	Elevated bilirubin	2	Lung disease	2	Anemia (hemoglobin < 10 g/dL)	1
					Thrombocytopenia (platelet < 15 k/µL)	1
					INR > 1.5	1
					Albumin < 3 g/dL	2
					Chronic lung disease	1
Primary endpoint	In-hospital mortality		10-years all-cause mortality		1-year all-cause mortality	
Score	Total = 0–12 Low risk ≤ 3 Intermediate risk = 4–5 High risk ≥ 6		Total = 0–12 Low risk ≤ 3 Intermediate risk = 4–6 High risk ≥ 7		Total = 0–16 No categorization	
C-statistic	>0.75		0.67		derivation cohort 0.712 validation cohort 0.729	

NYHA, New York Heart Association; HF, Heart Failure; LVEF, Left Ventricular Ejection Fraction; RV, Right Ventricle; GFR, Glomerular Filtration Rate; HR, Heart Rate; TR, Tricuspid Regurgitation; AST, Aspartate Aminotransferase; RVSP, Right Ventricular Systolic Pressure; CKD, Chronic Kidney Disease; INR, International Normalized Ratio.

**Table 2:** Summary of the most important transcatheter tricuspid valve repair and replacement devices.

	Device	Device Substances		Delivery
**TTVR**				
	EVOQUE	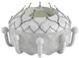	Self-expanding (nickel-titanium)	TF
	GATE	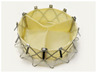	Self-expanding/Nitinol	TJ
	INTREPID	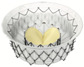	Dual-stent system	TF
	LuX-Valve	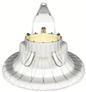	Self-expanding/Nitinol	TA
**Heterotropic Caval Valve Implantation**				
	TricValve	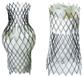	Self-expanding/Nitinol	TF
	Tricento	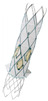	Self-expanding/Nitinol	TF
**TEER**	TriClip	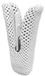	Paddles with nitinol skeleton	TF
	Pascal	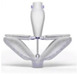	Paddles with nitinol skeleton	TF
**Annuloplasty**	Cardioband	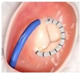	Sutureless contraction band covered by a polyester sleeve	TF

TTVR: transcatheter tricuspid valve replacement; TEER: transcatheter edge-to-edge repair; TF: transfemoral; TA: transapical; TJ: transjugular.
